# Optimizing immune checkpoint blockade in metastatic uveal melanoma: exploring the association of overall survival and the occurrence of adverse events

**DOI:** 10.3389/fimmu.2024.1395225

**Published:** 2024-06-10

**Authors:** Elias A. T. Koch, Anne Petzold, Edgar Dippel, Michael Erdmann, Anja Gesierich, Ralf Gutzmer, Jessica C. Hassel, Sebastian Haferkamp, Katharina C. Kähler, Nicole Kreuzberg, Ulrike Leiter, Carmen Loquai, Friedegund Meier, Markus Meissner, Peter Mohr, Claudia Pföhler, Farnaz Rahimi, Beatrice Schell, Patrick Terheyden, Kai-Martin Thoms, Selma Ugurel, Jens Ulrich, Jochen Utikal, Michael Weichenthal, Fabian Ziller, Carola Berking, Markus V. Heppt

**Affiliations:** ^1^Department of Dermatology, Uniklinikum Erlangen, Friedrich-Alexander-University Erlangen-Nürnberg (FAU), Erlangen, Germany; ^2^Comprehensive Cancer Center Erlangen-European Metropolitan Area of Nuremberg (CCC ER-EMN), Erlangen, Germany; ^3^Deutsches Zentrum Immuntherapie (DZI), Uniklinikum Erlangen, Friedrich-Alexander-University Erlangen-Nürnberg (FAU), Erlangen, Germany; ^4^Bavarian Cancer Research Center (BZKF), Uniklinikum Erlangen, Friedrich-Alexander-University Erlangen-Nürnberg (FAU), Erlangen, Germany; ^5^Department of Dermatology, Ludwigshafen Medical Center, Ludwigshafen, Germany; ^6^Department of Dermatology, University Hospital Würzburg, Würzburg, Germany; ^7^Skin Cancer Center Minden, Department of Dermatology, Mühlenkreiskliniken AöR, Ruhr University Bochum Campus Minden, Minden, Germany; ^8^Department of Dermatology and National Center for Tumor Diseases (NCT), NCT Heidelberg, a partnership between DKFZ and University Hospital Heidelberg, Medical Faculty Heidelberg, Heidelberg University, Heidelberg, Germany; ^9^Department of Dermatology, University Hospital Regensburg, Regensburg, Germany; ^10^Department of Dermatology, University Hospital Schleswig-Holstein, Kiel, Germany; ^11^Department of Dermatology and Venereology, Skin Cancer Center at the Center of Integrated Oncology (CIO) Köln Bonn, University Hospital of Cologne, Cologne, Germany; ^12^Department of Dermatology, Center for Dermatooncology, University Hospital Tübingen, Tübingen, Germany; ^13^Department of Dermatology, University Medical Center Mainz, Mainz, Germany; ^14^Department of Dermatology, Klinikum Bremen-Ost, Bremen, Germany; ^15^Skin Cancer Center at the University Cancer Centre Dresden and National Center for Tumor Diseases, Dresden, Germany; ^16^Department of Dermatology, University Hospital Carl Gustav Carus, TU Dresden, Dresden, Germany; ^17^Department of Dermatology, Venereology and Allergology, Goethe University, Frankfurt am Main, Germany; ^18^Department of Dermatology, Elbeklinikum, Buxtehude, Germany; ^19^Department of Dermatology, Saarland University Medical School, Homburg, Germany; ^20^Department of Dermatology and Allergy, Munich University Hospital (LMU), Munich, Germany; ^21^Department of Dermatology, SRH Wald-Klinikum Gera, Gera, Germany; ^22^Department of Dermatology, University of Lübeck, Lübeck, Germany; ^23^Department of Dermatology, University Medical Center Goettingen, Goettingen, Germany; ^24^Department of Dermatology, University Hospital Essen, University Duisburg-Essen, Essen, Germany; ^25^Department of Dermatology, Harzklinikum Dorothea Christiane Erxleben, Quedlinburg, Germany; ^26^Skin Cancer Unit, German Cancer Research Center (DKFZ) and Department of Dermatology, Venereology and Allergology, University Medical Center Mannheim, Ruprecht-Karl University of Heidelberg, Mannheim, Germany; ^27^DKFZ Hector Cancer Institute at the University Medical Center Mannheim, Mannheim, Germany; ^28^Department of Dermatology, DRK Krankenhaus Rabenstein, Chemnitz, Germany

**Keywords:** uveal melanoma, immune checkpoint blockade, PD-1, CTLA-4, immune-related, adverse events, toxicity

## Abstract

**Introduction:**

Despite recent advancements in the treatment of metastatic uveal melanoma (UM), the availability of further treatment options remains limited and the prognosis continues to be poor in many cases. In addition to tebentafusp, immune checkpoint blockade (ICB, PD-1 (+/-) CTLA-4 antibodies) is commonly used for metastatic UM, in particular in HLA-A 02:01-negative patients. However, ICB comes at the cost of potentially severe immune-related adverse events (irAE). Thus, the selection of patient groups that are more likely to benefit from ICB is desirable.

**Methods:**

In this analysis, 194 patients with metastatic UM undergoing ICB were included. Patients were recruited from German skin cancer sites and the ADOReg registry. To investigate the association of irAE occurrence with treatment response, progression-free survival (PFS), and overall survival (OS) two cohorts were compared: patients without irAE or grade 1/2 irAE (n=137) and patients with grade 3/4 irAE (n=57).

**Results:**

In the entire population, the median OS was 16.4 months, and the median PFS was 2.8 months. Patients with grade 3/4 irAE showed more favorable survival than patients without or grade 1/2 irAE (p=0.0071). IrAE occurred in 44.7% (87/194), and severe irAE in 29.4% (57/194) of patients. Interestingly, irColitis and irHepatitis were significantly associated with longer OS (p=0.0031 and p=0.011, respectively).

**Conclusions:**

This data may indicate an association between irAE and favorable survival outcomes in patients with metastatic UM undergoing ICB treatment and suggests that a reduced tolerance to tumor antigens could be linked to reduced tolerance to self-antigens.

## 1 Introduction

Uveal melanoma (UM) and cutaneous melanoma (CM) have a common origin in melanocytes, but they represent separate tumor entities. Unlike CM, UM harbors distinct mutations, an exceptionally low mutational burden (0.46 mutations per megabase), and an absence of the UV radiation mutational signature (1, 2). Robertson et al. have classified UM into four distinct prognostic groups based on TCGA data: disomy 3 accompanied by EIFAX mutations with a favorable prognosis, disomy 3 and SF3B1 mutation with an intermediate prognosis, and monosomy 3 with a poor prognosis. The latter can be further divided into two subsets, each exhibiting unique genomic aberrations and transcriptional features (3). Clinically, UM stands out as the most prevalent aggressive eye tumor among adults, yet it is an orphan tumor condition with an average incidence of around 5 per million in Europe and the USA (4). After initial diagnosis of the primary tumor, metastases are detectable in less than 4% of patients. However, over the further disease course, approximately 50% of UM patients develop metastases, depending on the genetic alterations of the tumor, primarily targeting the liver (4, 5). The follow-up after the initial diagnosis includes clinical and ophthalmological examinations, imaging of the liver, liver function tests, and tumor markers in peripheral blood (6). While liver function tests generally indicate a higher tumor burden, the primary detection of metastatic diseases typically occurs through ultrasound or magnetic resonance imaging of the liver (7). Blood-based tumor markers should ideally be capable of detecting metastatic diseases both at the initial diagnosis and during follow-up, suitable for monitoring the therapeutic response (8, 9). However, more specific biomarkers are needed for this purpose, which are not available to date. Once metastases emerge, the overall survival (OS) is still bleak (10, 11). The only therapy specifically approved for unresectable or metastatic UM by the Food and Drug Administration (FDA) and the European Medicines Agency (EMA) is currently tebentafusp (tebe) (12–14). In the pivotal trial, tebe-treated patients showed a median overall survival of 21.6 months after a 3-year follow-up, as opposed to 16.9 months in the control group, which received an investigator’s choice of pembrolizumab, ipilimumab, or dacarbazine (hazard ratio (HR) 0.68; 95% CI, 0.54 - 0.87), but not to dual checkpoint blockade (DCB = ipilimumab and nivolumab for up to four cycles, followed by nivolumab monotherapy) (15). Thus, Petzold et al. conducted a comprehensive meta-analysis of available systemic treatments, focusing on the comparison of tebe and DCB regarding OS and progression-free survival (PFS) (16). The study presented evidence that tebe is the most beneficial therapy option for metastatic UM in terms of OS. The median OS for tebe was 22.4 months, whereas DCB demonstrated a median OS of 15.7 months. The HR was 0.465 (95% CI: 0.276 – 0.781) for the matching-adjusted indirect comparison model and 0.641 (95% CI: 0.449–0.915) for the unadjusted model. Other treatment groups performed less favorably, with median OS ranging from 7.7 months (anti-CTLA-4 monotherapy) to 10.9 months (anti-PD-(L)-1 monotherapy). Moreover, a propensity score-weighted analysis comparing a prospective study of DCB with tebe also demonstrated a survival benefit for the latter (HR 0.52, 95% CI 0.35–0.78), but it is noteworthy that this analysis only encompassed one prospective trial of DCB, which exhibited a similarly low OS (17). However, tebe is accessible to only approximately 45–50% of Caucasian patients due to the HLA restriction to HLA*A02:01. Therefore, additional therapies like DCB remain important options for the treatment of metastatic UM, despite the small therapeutic benefit and the risk of severe immune-mediated adverse events (irAE). IrAE may potentially affect all organ systems due to a broad activation of the immune system, posing challenges in management, often requiring treatment interruption, systemic immunosuppression, and, in cases of intolerable toxicity, permanent treatment discontinuation (18–20). Grade 3–4 irAE occur in 55% of patients treated with nivolumab plus ipilimumab in CM (21). This rate of severe irAE aligns with reports in UM, where grade 3/4 toxicity occurs in up to 57.7% (22–25). The correlation between survival benefit and the occurrence of irAE in CM has been investigated. There is evidence suggesting that reduced tolerance to tumor antigens is linked to reduced tolerance to self-antigens (26–30). However, an association between the occurrence of irAE and the outcomes of immune checkpoint blockade (ICB) in metastatic UM has not been investigated yet.

## 2 Materials and methods

### 2.1 Patient population and study design

We performed a retrospective multi-center explorative analysis. Inclusion criteria were histologically confirmed stage IV UM, a follow-up time of at least three months after the start of therapy, and application of any type of ICB treatment (ipilimumab, nivolumab, pembrolizumab, DCB) between 2013 and 2021. A total of 194 patients were included and divided into two cohorts. Cohort A comprised patients without or grade 1/2 irAE (n=137) and cohort B patients with grade 3–5 irAE undergoing ICB treatment (n=57). Additionally, subgroup analyses were performed for patients without any irAE and those with permanent discontinuation due to treatment-induced toxicity.

Clinical data and the treatment outcomes of interest were extracted from the original patient records from 16 German skin cancer centers (Erlangen n=59, Tübingen n=20, München n=18, Mainz n=7, Kiel n=5, Mannheim n=5, Frankfurt n=4, Heidelberg n=4, Dresden n=3, Köln n=3, Göttingen n=2, Homburg n=2, Ludwigshafen n=2, Lübeck n=2, Würzburg n=2, Essen n=1) as well as from the ADOReg registry of the German Dermatologic Cooperative Oncology Group (DeCOG, n=55). The data were collected and merged into a central database before analysis. The ADOReg registry is a large prospective clinical database in the field of dermatologic oncology collecting data to generate high-quality real-world evidence. This study was approved by the scientific board of the registry and by the institutional review board of the medical faculty of the Munich University Hospital (approval number 413–16 UE). Furthermore, it was conducted following the principles of the Helsinki Declaration in its current version.

### 2.2 Data collection and treatment outcomes

The clinical data recorded at baseline comprised demographics with sex, age, number of organ systems affected by metastasis, and date of death or last documented patient contact. At the date of ICB start the Eastern Cooperative Oncology Group (ECOG) performance status and serum lactate dehydrogenase (LDH) were collected from patient charts and analyzed for their prognostic value. We recorded ICB start and end date, time to progression, and best response evaluation based on the RECIST criteria version 1.1. The best radiologic response to treatment was assessed by the site investigators and indicated as complete response (CR), partial response (PR), stable disease (SD), or progressive disease (PD) based on the RECIST criteria version 1.1 (31). CR and PR were summarized as objective response rates (ORR) and CR, PR, and SD as disease control rate (DCR). In all cases, patients were treated until disease progression or until the development of unacceptable toxicity for which ICB was permanently discontinued. In addition, we summarized as “other metastases” any metastases besides liver, bone, pulmonary, central nervous system (CNS), lymph node, connective tissue, and skin metastases.

IrAE were retrospectively assessed by the site investigators based on the patient records and clinical outcomes according to the Common Terminology Criteria for Adverse Events (CTCAE) v5.0 published by the National Institutes of Health in 2017.

### 2.3 Statistical analyses

OS was calculated as the time from the diagnosis of stage IV UM until melanoma-specific or treatment-related death. The PFS was determined as the time from treatment start until disease progression. Time-to-event analyses were calculated where death or progression was considered as an event. If neither occurred or if patients were lost to follow-up, the date of the last documented presentation was used as a censored observation.

The survival and progression probabilities were indicated with the Kaplan-Meier method and log-rank tests were performed for comparing these probabilities in the two groups. Furthermore, χ^2^ and t-tests were conducted (1) to show the comparability of the two cohorts and (2) to compare the response rates. In case of significantly different baseline characteristics, we conducted a multivariate COX model that calculated the HR adjusting for these parameters. Subsequently, we compared the multivariate COX model with the univariate models to identify potentially significant confounders influencing the survival outcome. In all cases, two-tailed p-values were calculated and considered significant with values p < 0.05. Patients with missing values for a given variable were excluded. No imputation of missing data was performed. All analyses were carried out with the software R (https://www.r-project.org/) using the packages “survival” and “survminer”.

## 3 Results

### 3.1 Characteristics of the study population

A total of 194 (100%) patients with metastatic UM who underwent therapy with ICB were included (**Table 1**). The study population was divided into two cohorts to find correlations between the occurrence and severity of irAE and prognosis. Cohort A included patients without or low-grade 1/2 irAE (n=137) and cohort B patients with high-grade 3/4 irAE undergoing ICB treatment (n=57). A median of two organ systems was affected by metastasis, predominantly liver (91.8%), lung (46.9%), and bones (26.8%). 49.5% had an ECOG status of 0. Serum LDH was within normal limits in 48% when ICB was initiated. The two cohorts exhibited significant differences in the administered ICB substance (p<0.001 for anti-PD1 and DCB), as well as in the number of affected organ systems (p=0.0076). To assess whether these factors confounded the survival outcome in the two cohorts, we conducted a multivariate Cox model that adjusted for (1) the ICB substance (DCB versus other ICB treatments) and (2) the number of affected organ systems (≤ 2 versus >2 affected organ systems). We compared this multivariate model against the univariate Cox model, which calculated the HR of cohort A versus B without any adjustment (see **Table 2**). The univariate model showed an HR of 0.50, while the multivariate model showed an HR of 0.48. The likelihood ratio test indicated no significant difference between these models (p=0.55), suggesting that these factors did not exert a crucial influence on survival in this study. Thus, we could proceed to analyze the survival difference between the two cohorts further.

**Table 1:** Characteristics of the study population.

Category	unit	Total (N=194)	Cohort A (n=137)	Cohort B (n=57)	Test (A vs B)
Sex	women	97 (50.0%)	73 (53.3%)	24 (42.1%)	p=0.21
	men	97 (50.0%)	64 (46.7%)	33 (57.9%)	
Age at start ICB	Median (range) in years	65.1 (17.7–87.6)	65.6 (17.7–85.4)	64.2 (36.8–87.6)	p=0.68
LDH	not elevated	48 (%)	32 (23.4%)	16 (28.1%)	p=0.23
	elevated	93 (%)	70 (51.1%)	23 (40.4%)	
	NA	53 (%)	35 (25.5%)	18 (31.5%)	
ECOG	0	96 (49.5%)	63 (46.0%)	33 (57.9%)	p=0.18
	1	21 (10.8%)	19 (13.9%)	2 (3.5%)	
	2	4 (2.1%)	3 (2.2%)	1 (1.8%)	
	3	2 (1.0%)	2 (1.5%)	0 (0.0%)	
	NA	71 (36.6%)	50 (36.5%)	21 (36.8%)	
Number of organ systems affected by metastases	Median; range	2 (1–8)	3 (1–8)	2 (1–7)	p=0.0076
Localization of the affected organ systems by metastases	Liver:	178 (91.8%)	130 (94.9%)	48 (84.2%)	p=0.03
	Pulmonary:	91 (46.9%)	68 (49.6%)	23 (40.4%)	
	Bone:	52 (26.8%)	45 (32.8%)	7 (12.3%)	
	CNS:	27 (13.9%)	18 (13.1%)	9 (15.8%)	
	Lymph node:	44 (22.7%)	35 (25.5%)	9 (15.8%)	
	Connective tissue:	9 (4.6%)	8 (5.8%)	1 (1.8%)	
	Skin:	26 (13.4%)	18 (13.1%)	8 (14.0%)	
	Disseminated:	10 (5.2%)	9 (6.6%)	1 (1.8%)	
	Other:	55 (28.4%)	43 (31.4%)	12 (21.1%)	
	NA:	4 (2.1%)	2 (1.5%)	2 (3.5%)	
ICB as first-line therapy	N and %	160 (82.5%)	117 (85.4%)	43 (75.4%)	p=0.15
Number of pretreatments	Median (range)	0 (0–3)	0 (0–3)	0 (0–2)	p=0.23
ICB substance	All	193 (99.5%)	136 (99.3%)	57 (100%)	p=1
	Anti-PD1 (nivolumab/pembrolizumab)	56 (28.9%)	51 (37.2%)	5 (8.8%)	p<0.001
	Anti-CTLA4 (ipilimumab)	15 (7.7%)	12 (8.8%)	3 (5.3%)	p=0.59
	Dual Checkpoint Blockade	122 (62.9%)	73 (53.3%)	49 (86.0%)	p<0.001
	NA	1 (0.5%)	1 (0.7%)	0 (0.0%)	

NA, not available; ICB, immune checkpoint blockade; CNS, central nervous system. Cohort A = patients without or low-grade (1/2) irAE. Cohort B = patients with high-grade (3/4) irAE.

**Table 2:** Univariate COX model versus multivariate COX model demonstrating that the occurrence of severe irAE was a significant factor for overall survival (OS) independent of the application of DCB and the number of affected organ systems.

	Parameter	Category	Hazard Ratio (95% CI)	p-value	likelihood ratio test
Univariate COX model	irAE grade	≤2 (cohort A) 3 or 4 (cohort B)	0.50 (0.30–0.83) 2.00	0.007	Univariate vs. multivariate COX model p=0.55
Multivariate COX model	irAE grade	≤2 (cohort A) 3 or 4 (cohort B)	0.48 (0.28–0.82) 2.10	0.007	
	ICB treatment	other ICB DCB	0.89 (0.57–1.39) 1.13	0.60	
	Number of affected organ systems	≤2 >2	0.79 (0.50–1.24) 1.27	0.31	

ICB, immune checkpoint blockade; DCB, dual checkpoint blockade.

### 3.2 Treatment response and survival outcomes

The median OS of the entire population was 16.4 months (95% CI 14.1–23.8). The median PFS in stage IV disease after ICB was 2.8 months (95% CI 2.4–3.0). The majority of patients received DCB (62.9%, n=122) while anti-PD1 and anti-CTLA-4 were applied in 28.9% (n=56) and 7.7% (n=15), respectively. 82.5% (160/194) received ICB as first-line treatment.

**Figure 1:**
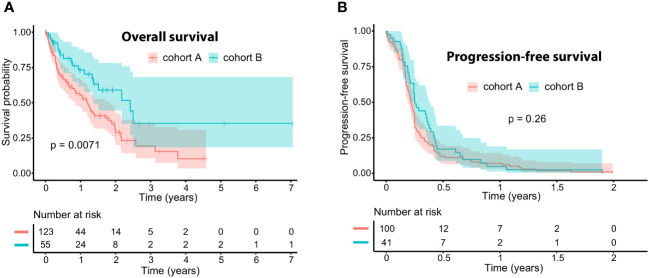
Kaplan-Meier curves for **(A)** overall survival (OS) and **(B)** progression-free survival (PFS) to immune checkpoint blockade (ICB), comparing patients with no or mild adverse events (cohort A, red) versus patients with severe adverse events (cohort B, turquoise). The median OS is 14.5 months (95% CI: 10.3–21.8) for cohort A and 29.0 months (95% CI: 16.4 – NR) for cohort B. The median progression-free survival is 2.6 months (95% CI: 2.3–3.0) for cohort A and 3.0 months (95% CI: 2.9 – 4.5) for cohort B. For OS **(A)**, there were 14 missing data points regarding the start date of ICB treatment or the date of last contact/death in cohort A, leaving 123 patients at risk initially. In cohort B, 2 data points were missing regarding the date of last contact or death, resulting in 55 patients at risk at the outset. For PFS **(B)**, there were 37 progression dates unavailable in cohort A, leaving 100 patients at risk initially, while cohort B had 16 missing data points, resulting in 41 patients at risk initially.

**Figure 2:**
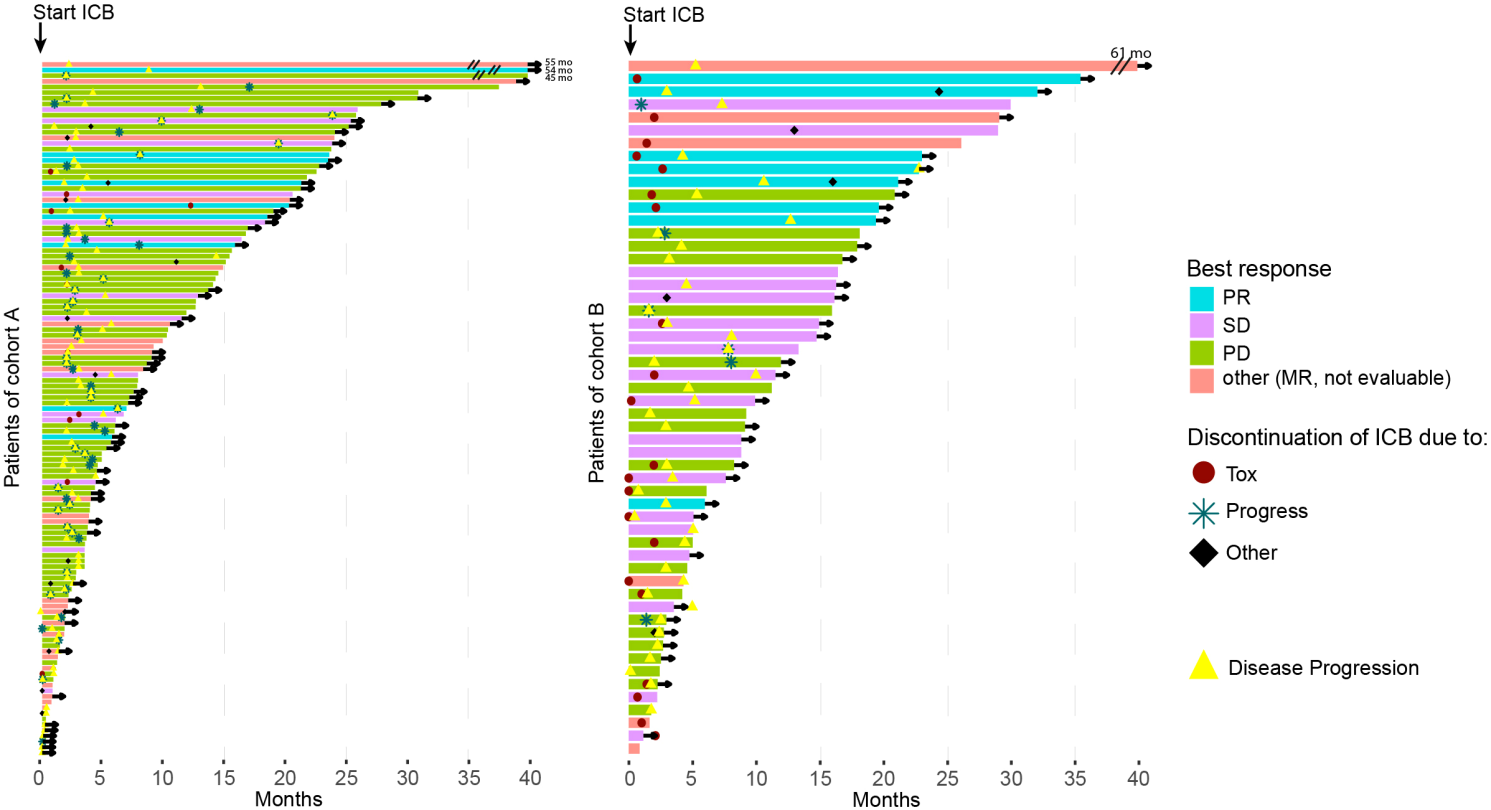
Swimmer plots for Cohort A (left) and B (right) illustrate the overall survival (OS) for each patient. The color represents the best response to immune checkpoint blockade (ICB) for the patient, while symbols depict the reason for the termination of ICB treatment. The yellow triangle marks the point of tumor progression, and if the patient is censored, an arrow is drawn. For visual clarity, the OS duration of patients at the top is shortened (indicated by two black lines, with the actual OS written behind in months).

The median OS differed significantly between the cohorts: (A) median OS 14.5 months (95%-CI 10.3–21.8) vs. (B) median OS 29 months (95%-CI 16.4-NR, p=0.0071). In contrast, the median PFS only differed slightly: (A) median PFS 2.6 months (95%-CI 2.3–3) vs. (B) 3 months (95%-CI 2.9–4.5; p=0.26). Details are presented in the Kaplan-Meier curves in **Figure 1**. Further information on the survival times of both cohorts is presented in the swimmer plots (**Figure 2**). The ORR for all ICB regimens was 11%, 13.1% for DCB, and 8.4% for anti-PD1 (**Table 3**). Notably, the DCR was considerably increased in cohort B for all ICB and DCB treatments (p=0.001 and p=0.07, respectively). The ORR to anti-PD-1 was significantly lower in cohort A (4.7% vs. 50%, p=0.03).

**Table 3:** Response rates to ICB according to ICB substance.

ICB All	Total	Cohort A	Cohort B	Test (A vs B)
CR	0.6% (1/164)	0% (0/111)	1.9% (1/53)	p=1
PR	10.4% (17/164)	8.1% (9/111)	15.1% (8/53)	p=0.27
SD	25% (41/164)	18.9% (21/111)	37.7% (20/53)	p=0.016
PD	59.8% (98/164)	67.6% (/111)	43.4% (23/53)	p=0.15
ORR	11% (18/164)	8.1% (9/111)	16.9% (9/53)	p=0.15
DCR	36% (59/164)	27% (30/111)	54.7% (29/53)	p=0.001
Anti-PD-1	Total	Cohort A	Cohort B	Test (A vs B)
CR	0% (0/47)	0% (0/43)	0% (0/4)	
PR	8.5% (4/47)	4.7% (2/43)	50% (2/4)	p=0.03
SD	19.1% (9/47)	18.6% (8/43)	25% (1/4)	p=1
PD	68.1% (32/47)	72.1% (31/43)	25% (1/4)	p=0.17
ORR	8.5% (4/47)	4.7% (2/43)	50% (2/4)	p=0.03
DCR	27.7% (13/47)	23.3% (10/43)	75% (3/4)	p=0.1
DCB	Total	Cohort A	Cohort B	Test (A vs B)
CR	0.9% (1/106)	0% (0/58)	2.1% (1/48)	p=0.92
PR	12.3% (13/106)	12.1% (7/58)	12.5% (6/48)	p=1
SD	28.3% (30/106)	20.7% (12/58)	37.5% (18/48)	p=0.09
PD	13.2% (14/106)	60.3% (35/58)	45.8% (22/48)	p=0.2
ORR	13.2% (14/106)	12.1% (7/58)	14.6% (7/48)	p=1
DCR	44/106 = 41.5%	32.8% (19/58)	52.1% (25/48)	p=0.07

CR, complete response; PR, partial response; SD, stable disease; PD, progressive disease; ORR, objective response rate; DCR, disease control rate; ICB, immune checkpoint blockade; DCB, dual checkpoint blockade. Missing data were not considered in the table.

### 3.3 Characterization of irAE

**Figure 3:**
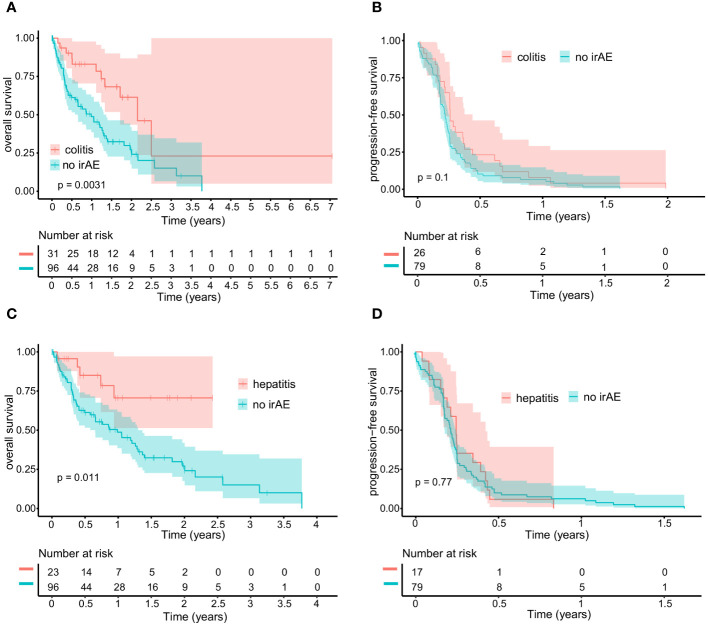
Kaplan-Meier curves comparing patients with irColitis **(A, B)** or irHepatitis **(C, D)** to patients without any irAE. For irColitis patients, the median overall survival (OS) is 25.9 months (95% CI: 20.6 - NR), and the median progression-free survival (PFS) is 3.0 months (95% CI: 2.5 - 5.2). For irHepatitis patients, the median OS is not reached, and the median PFS is 2.9 months (95% CI: 2.3 - 5.1). For patients without any irAE, the median OS is 11.9 months (95% CI: 7.8 – 16.4), and the median PFS is 2.5 months (95% CI: 2.1 – 2.9). For OS **(A)**, there were three dates of last contact/death unavailable in the group of patients with irColitis, leaving 31 patients at risk initially, while the group of patients without irAE had 11 missing data points, resulting in 96 patients at risk in the beginning. For PFS **(B)**, there were eight missing data points regarding the date of progression in the group with irColitis patients, leaving 26 patients at risk initially, while 37 data points were missing in the cohort of patients without irAE, resulting in 79 patients at risk initially. For OS **(C)**, there were no missing data points in the group of patients with irHepatitis, while the group without irAE had 11 missing data points, resulting in 96 patients at risk initially. For PFS **(D)**, there were six progression dates unavailable for the cohort with irHepatitis, leaving 17 patients at risk at the outset, while 37 data points were missing in the cohort without irAE, resulting in 79 patients at risk initially.

A total of 160 irAE were reported in 87 (44.7%) patients (**Supplementary Table S1**). Of all events, 108 irAE were graded as severe (grade 3–5) and were observed in 57 patients. The treatment was permanently discontinued in 41 cases due to unacceptable toxicity. No death occurred due to toxicity, one death occurred in cohort A during treatment, associated with disease progression. The most common events were irColitis (n=36), irHepatitis (n=24), irThyroiditis (n=14), irHypophysitis (n=13), irMyalgia with irMyositis (n=8), and cutaneous irAE (n=7). Patients with irColitis and irHepatitis showed significantly improved OS compared to those without any irAE, independent of their severity (p=0.0031 and p=0.011, respectively; **Figure 3**). A comparison of patients with irColitis, irHepatitis, other irAE, and no irAE showed also a significant difference (p<0.001, **Supplementary Figure S1**). In a further subgroup analysis, patients with permanent treatment discontinuation due to immune-related toxicity (40/41 were evaluable) showed a trend toward prolonged OS, albeit without statistical significance (p=0.075, **Supplementary Figure S2**).

## 4 Discussion

In this multicenter study, including a comparably large number of patients with metastatic UM (n=194), the association between irAE of ICB treatment and clinical outcomes was investigated. Patients with severe irAE (grade 3–5, cohort B) had an improved OS (29 months vs 14.5 months, p=0.006, HR=0.5) compared to those with none, mild or moderate irAE (grade 0–2, cohort A). Cohort B comprised a higher number of patients undergoing DCB (p<0.001), which is associated with a significantly higher incidence of severe irAE and is more prone to affecting multiple organs when compared to single ICB (32). In addition, DCB has an improved ORR compared to single anti-PD-1 therapy (13.2% vs 8.5%, respectively). By comparing the multivariate COX model with the univariate model and assessing the difference in HR, we have shown that the incidence of severe irAE was linked to extended OS regardless of the administration of DCB and the number of affected organ systems (likelihood ratio test (p=0.55)). Ultimately, it suggests that severe irAE may reflect a response to treatment and translate into better survival outcomes in patients with metastatic UM. Associations between irAE and treatment response in other tumor types have been reported, but evaluations in metastatic UM have not been performed to date. A retrospective study in patients with CM undergoing single ICB or DCB demonstrated improved OS in patients with irAE of any grade (26), which is in line with previous reports (33, 34). Further reports exist on the association between survival benefits in patients with non-small cell lung cancer and other tumor types undergoing ICB and the occurrence of irAE (35–38). Specifically, cutaneous irAE were associated with longer OS in advanced cancer patients (39, 40). Additionally, reports have shown that irColitis was associated with improved OS, consistent with our results indicating a significant association between both irColitis and irHepatitis and OS (41). For other irAE, the sample size was too small to draw sufficient conclusions in this analysis.

In a meta-analysis comprising 52 studies and involving a total of 9,156 patients, it was demonstrated that there is a 3-fold higher response rate in tumors, significantly improved OS and PFS in cancer patients who received ICB and experienced irAE compared to those who did not have any irAE (42). Notably, to minimize bias related to the duration of ICB, an extended analysis focused on patients with at least a 6-week exposure to ICB. The results also indicated that the occurrence of any grade irAE was positively associated with survival benefits (PFS and OS). The authors suggested that this effect was mediated through bystander effects of re-activated T-cells (42). Therefore, it is plausible that patients benefiting more from ICB are likely to experience severe autoimmune toxicities. Notably, another meta-analysis including 51 studies and several tumor entities demonstrated that irAE were associated with increased OS, PFS, and ORR, and grade 3 or higher irAE showed also higher ORR but worse OS (30).

Short-time use of systemic corticosteroids to manage irAE does not negatively affect antitumor responses (27, 29, 43). Notably, in a study investigating patients with CM under first-line DCB, second-line immunosuppression for irAE demonstrated an association with reduced PFS and OS compared to those whose irAE were managed with systemic corticosteroids only (44). Most of these patients received anti-TNF (58%) as a second-line immunosuppressant. However, these results emphasize the relevance of evaluating the impacts of immunosuppression for managing irAE.

An analysis of the prospective skin cancer registry ADOREG revealed that patients with advanced CM undergoing ICB and with brain metastases received immunosuppression more frequently compared to patients without brain metastases (45). Among these patients, those receiving concomitant immunosuppression before the start of ICB showed worse OS. However, initiation of immunosuppression within 30 days after the start of ICB, mostly due to irAE, did not affect the efficacy of ICB (45).

In our study population, we detected a median OS of 16.4 months (95% CI: 14.1 - 23.8) and a median PFS to any ICB of 2.8 months (95% CI: 2.4 - 3.0). The median OS of 16.4 months was higher compared to studies completed before the ICB era and lower compared to the pivotal trials of tebe (11, 15, 46–48). The ORR to DCB of 13.1% remained poor with no significant differences between the cohorts and a slight tendency toward worse ORR to DCB in patients with none or mild to moderate irAE. The response rate to DCB is consistent with published retrospective studies by us and others, reporting an ORR of 11.6–16.7% (5, 22, 23, 49). Additionally, it aligns with findings from published prospective studies demonstrating an ORR of 11.5% and 18% (24, 25).

Limitations of this study are its retrospective design and the resulting selection bias due to the missing randomization. Another limitation lies in the validity of the data, particularly in the reporting of irAE, as it heavily relies on the documentation practices of the center and the investigator’s discretion and expertise in assessing irAE as treatment-related events. Thus, irAE may be underreported compared to prospective studies and the possibility of lead-time bias concerning the ORR exists since the precise timing of irAE was not documented. Further limitations are, that the immunosuppression for managing irAE was not assessed and subsequent treatments were not considered, as second-line immunosuppression and other treatments might impact OS.

## 5 Conclusions

Our study underscores the correlation between irAE and survival outcomes in patients with metastatic UM receiving ICB treatment. It suggests that the incidence of severe treatment-related toxicity is associated with enhanced clinical benefits.

## Data availability statement

The original contributions presented in the study are included in the article/**Supplementary Material**. Further inquiries can be directed to the corresponding author.

## Ethics statement

This study was approved by the institutional review board of the medical faculty of the Munich University Hospital (approval number 413-16 UE) and was conducted in accordance with the principles of the Helsinki Declaration in its current version. The studies were conducted in accordance with the local legislation and institutional requirements. The ethics committee/institutional review board waived the requirement of written informed consent for participation from the participants or the participants’ legal guardians/next of kin for this study due to its retrospective design.

## Author contributions

EATK: Conceptualization, Data curation, Investigation, Methodology, Project administration, Writing – original draft, Writing – review & editing. AP: Formal Analysis, Visualization, Writing – review & editing. ED: Data curation, Writing – review & editing. ME: Data curation, Writing – review & editing. AG: Data curation, Writing – review & editing. RG: Data curation, Writing – review & editing. JCH: Data curation, Visualization, Writing – review & editing. SH: Data curation, Writing – review & editing. KCK: Data curation, Writing – review & editing. NK: Data curation, Writing – review & editing. UL: Data curation, Writing – review & editing. CL: Data curation, Writing – review & editing. FM: Data curation, Writing – review & editing. MM: Data curation, Writing – review & editing. PM: Data curation, Writing – review & editing. CP: Data curation, Writing – review & editing. FR: Data curation, Writing – review & editing. BS: Data curation, Writing – review & editing. PT: Data curation, Writing – review & editing. K-MT: Data curation, Writing – review & editing. SU: Data curation, Writing – review & editing. JUl: Data curation, Writing – review & editing. JUt: Data curation, Writing – review & editing. MW: Data curation, Writing – review & editing. FZ: Data curation, Writing – review & editing. CB: Data curation, Project administration, Resources, Writing – review & editing. MVH: Conceptualization, Data curation, Methodology, Project administration, Supervision, Writing – review & editing.

## Supplementary material

Supplementary Figure 1 Kaplan-Meier curves for **(A)** overall survival (OS) and **(B)** progression-free survival (PFS) to immune checkpoint blockade (ICB), comparing patients with irColitis (red), irHepatitis (green), other irAE (turquoise) and no irAE (purple). The median OS is 25.9 months (95% CI: 20-NR) for irColitis, NR for irHepatitis, 23.8 months (95% CI: 16.4-NR) for other irAE, and 11.9 months (95% CI: 7.8–16.4) for no irAE. The median PFS is 3 months (95% CI: 2.5–5.2) for irColitis, 2.9 (95% CI: 2.3–5.1) for irHepatitis, 2.9 months (95% CI: 2.3–4.5) for other irAE, and 2.5 months (95% CI: 2.1–2.9) for no irAE. The missing data points related to OS **(A)** were as follows: in the irColitis group, there were 3, leaving 31 patients at risk initially; in the irHepatitis group, there were 0; in the group experiencing other irAE, there was 1, leaving 34 patients at risk initially; and in the group without irAE, there were 11, resulting in 96 patients at risk in the beginning. For PFS **(B)**, there were 8 missing data points for irColitis, leaving 26 patients at risk initially; for irHepatitis, there were 6, resulting in 17 patients at risk initially; for patients with other irAE, there were 11, resulting in 24 patients at risk in the beginning; and for the group without irAE, there were 37 missing data points, leaving 79 patients initially.

Supplementary Figure 2 Kaplan-Meier curves for **(A)** overall survival (OS) and **(B)** progression-free survival (PFS) to immune checkpoint blockade (ICB), comparing patients with permanent treatment discontinuation due to toxicity (red) and other patients (turquoise). The median OS is 22.6 months (95% CI: 14.9-NR) versus 15.4 months (95% CI: 11.9–23.7), respectively. The median PFS is 3 months (95% CI: 2.3–4.5) versus 2.6 (95% CI: 2.4–3), respectively. For OS **(A)**, there was one missing data point in the cohort with toxicity, leaving 40 patients at risk initially, while 15 data points were unavailable in the group without discontinuation due to toxicity, resulting in 138 patients at risk at the outset. Regarding PFS **(B)**, 11 data points were unavailable in the group with toxicity, leaving 30 patients at risk initially, while 42 data points were missing in the cohort without toxicity, resulting in 111 patients at risk in the beginning.

Supplementary Table 1 Occurrence of irAE.
