# Supplementary figures and images for: Optimizing immune checkpoint blockade in metastatic uveal melanoma: exploring the association of overall survival and the occurrence of adverse events
Source: Front Immunol. 2024 Jun 10;15:1395225. doi: 10.3389/fimmu.2024.1395225 (PMC11194381; doi:10.3389/fimmu.2024.1395225)

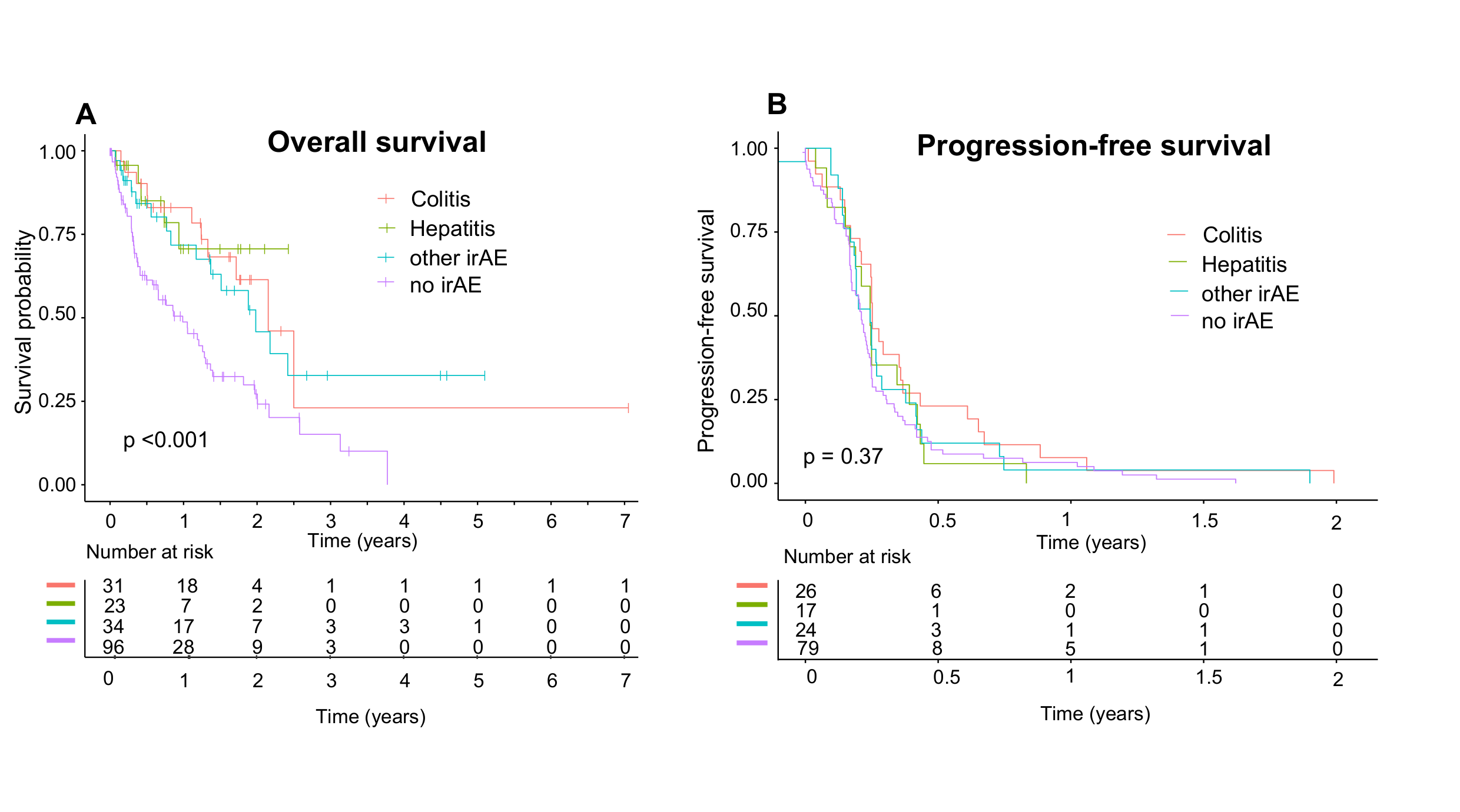

Supplement: Supplementary Figure 1 — Kaplan-Meier curves for (A) overall survival (OS) and (B) progression-free survival (PFS) to immune checkpoint blockade (ICB), comparing patients with irColitis (red), irHepatitis (green), other irAE (turquoise) and no irAE (purple). The median OS is 25.9 months (95% CI: 20-NR) for irColitis, NR for irHepatitis, 23.8 months (95% CI: 16.4-NR) for other irAE, and 11.9 months (95% CI: 7.8–16.4) for no irAE. The median PFS is 3 months (95% CI: 2.5–5.2) for irColitis, 2.9 (95% CI: 2.3–5.1) for irHepatitis, 2.9 months (95% CI: 2.3–4.5) for other irAE, and 2.5 months (95% CI: 2.1–2.9) for no irAE. The missing data points related to OS (A) were as follows: in the irColitis group, there were 3, leaving 31 patients at risk initially; in the irHepatitis group, there were 0; in the group experiencing other irAE, there was 1, leaving 34 patients at risk initially; and in the group without irAE, there were 11, resulting in 96 patients at risk in the beginning. For PFS (B), there were 8 missing data points for irColitis, leaving 26 patients at risk initially; for irHepatitis, there were 6, resulting in 17 patients at risk initially; for patients with other irAE, there were 11, resulting in 24 patients at risk in the beginning; and for the group without irAE, there were 37 missing data points, leaving 79 patients initially. [file Image_1.tiff]

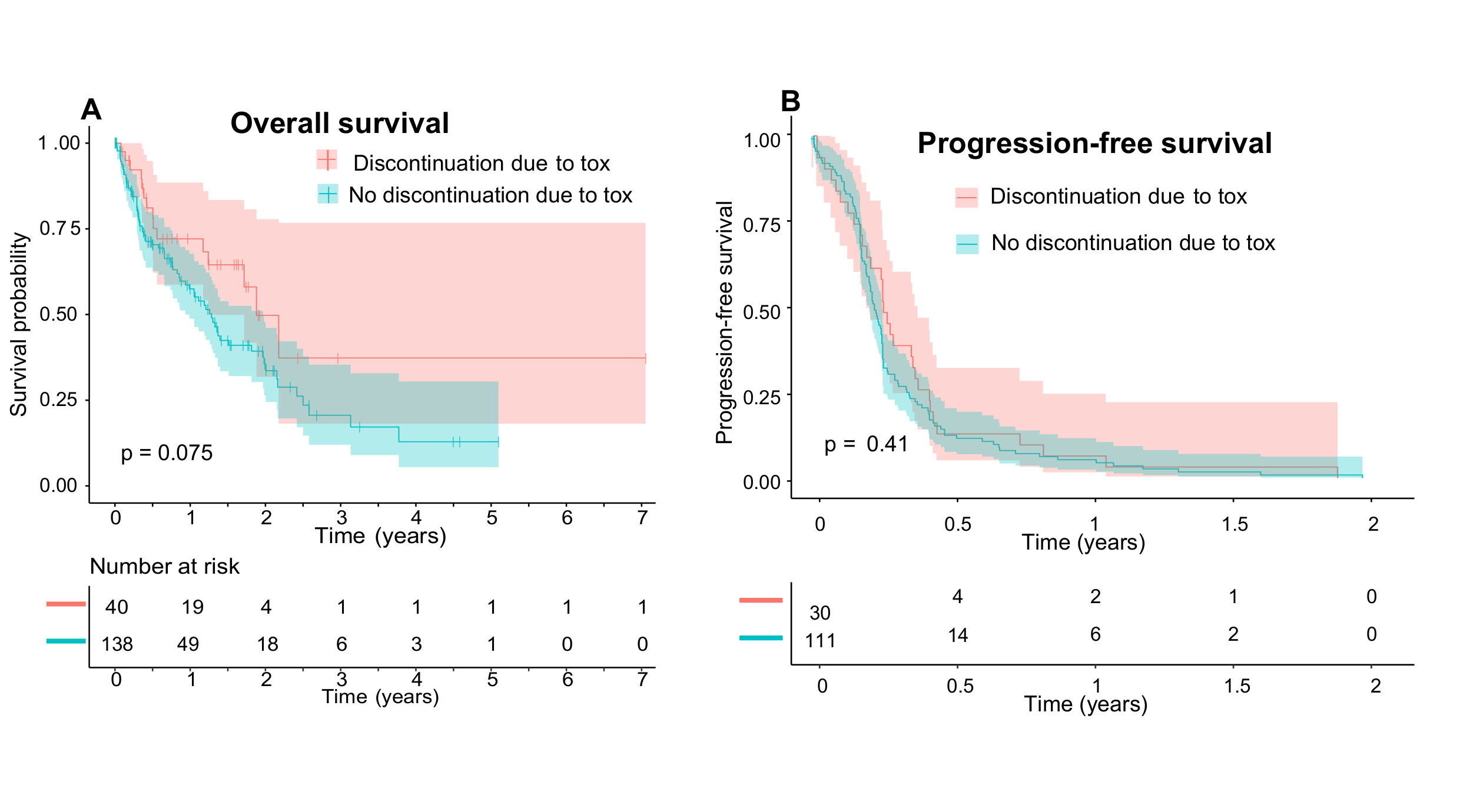

Supplement: Supplementary Figure 2 — Kaplan-Meier curves for (A) overall survival (OS) and (B) progression-free survival (PFS) to immune checkpoint blockade (ICB), comparing patients with permanent treatment discontinuation due to toxicity (red) and other patients (turquoise). The median OS is 22.6 months (95% CI: 14.9-NR) versus 15.4 months (95% CI: 11.9–23.7), respectively. The median PFS is 3 months (95% CI: 2.3–4.5) versus 2.6 (95% CI: 2.4–3), respectively. For OS (A), there was one missing data point in the cohort with toxicity, leaving 40 patients at risk initially, while 15 data points were unavailable in the group without discontinuation due to toxicity, resulting in 138 patients at risk at the outset. Regarding PFS (B), 11 data points were unavailable in the group with toxicity, leaving 30 patients at risk initially, while 42 data points were missing in the cohort without toxicity, resulting in 111 patients at risk in the beginning. [file Image_2.tiff]
