# Supplementary material for: Optimizing immune checkpoint blockade in metastatic uveal melanoma: exploring the association of overall survival and the occurrence of adverse events
Source: Front Immunol. 2024 Jun 10;15:1395225. doi: 10.3389/fimmu.2024.1395225 (PMC11194381; doi:10.3389/fimmu.2024.1395225)
Supplement: Supplementary Table 1 — Occurrence of irAE. [file Table_1.docx]

**Supplementary Table 1:** Occurrence of irAE

| Adverse events (AE) | Total (160 AE) | Cohort A (58 AE) | Cohort B (108 AE) |
| --- | --- | --- | --- |
| Colitis | 36 (18.6%) | 11 (8.0%) | 25 (43.9%) |
| Hepatitis | 24 (12.4%) | 6 (4.4%) | 18 (31.6%) |
| Thyreoiditis | 14 (7.2%) | 5 (3.6%) | 9 (15.8%) |
| Hypophysitis | 13 (6.7%) | 2 (1.5%) | 11 (19.3%) |
| Pancreatitis | 4 (2.1%) | 0 (0.0%) | 4 (7.0%) |
| Cutaneous AE | 7 (3.6%) | 5 (3.6%) | 2 (3.5%) |
| Myositis | 8 (4.1%) | 3 (2.2%) | 5 (8.8%) |
| Myocarditis | 4 (2.1%) | 1 (0.7%) | 3 (5.3%) |
| General disorders (avolition, fever, night sweats) | 7 (3.6%) | 3 (2.2%) | 4 (7.0%) |
| Pruritus | 3 (1.5%) | 2 (1.5%) | 1 (1.8%) |
| Uveitis | 2 (1.0%) | 1 (0.7%) | 1 (1.8%) |
| Peripheral neuropathies | 3 (1.5%) | 1 (0.7%) | 2 (3.5%) |
| Fatigue | 2 (1.0%) | 1 (0.7%) | 1 (1.8%) |
| Anemia | 2 (1.0%) | 0 (0.0%) | 2 (3.5%) |
| Nephritis | 2 (1.0%) | 0 (0.0%) | 2 (3.5%) |
| Gastritis | 2 (1.0%) | 1 (0.7%) | 1 (1.8%) |
| Adrenal Insufficiency | 2 (1.0%) | 1 (0.7%) | 1 (1.8%) |
| Pneumonitis | 5 (2.6%) | 1 (0.7%) | 4 (7.0%) |
| Diabetes | 2 (1.0%) | 0 (0.0%) | 2 (3.5%) |
| Infusion associated symptoms | 2 (1.0%) | 1 (0.7%) | 1 (1.8%) |
| Alveolitis | 1 (0.5%) | 0 (0.0%) | 1 (1.8%) |
| Vertigo | 1 (0.5%) | 1 (0.7%) | 0 (0.0%) |
| Impaired vision | 1 (0.5%) | 1 (0.7%) | 0 (0.0%) |
| Endocrinopathy | 1 (0.5%) | 0 (0.0%) | 1 (1.8%) |
| Autoimmune disorder | 1 (0.5%) | 1 (0.7%) | 0 (0.0%) |
| Pulmonary embolism | 1 (0.5%) | 0 (0.0%) | 1 (1.8%) |
| GI bleeding | 1 (0.5%) | 0 (0.0%) | 1 (1.8%) |
| Hearing impairment | 1 (0.5%) | 1 (0.7%) | 0 (0.0%) |
| Dry chesty cough | 1 (0.5%) | 1(0.7%) | 0 (0.0%) |
| Hyperhidrosis | 1 (0.5%) | 1 (0.7%) | 0 (0.0%) |
| Death | 1 (0.5%) | 0 (0.0%) | 1 (1.8%) |
| Sialadenitis | 1 (0.5%) | 1 (0.7%) | 0 (0.0%) |
| Anosmia | 1 (0.5%) | 0 (0.0%) | 1 (1.8%) |
| Intracerebral bleeding | 2 (1.0%) | 0 (0.0%) | 2 (3.5%) |
| Deep venous thrombosis | 1 (0.5%) | 0 (0.0%) | 1 (1.8%) |
